# British Red Cross Society

**Published:** 1906-06-02

**Authors:** 


### BRITISH RED CROSS SOCIETY.

### MESSAGE FROM THE PRINCE OF WALES.

Lord Rothschild presided at the Mansion House on Wednesday, May 23, at a meeting of the British Red Cross Society, the Lord Mayor, who should have presided, being engaged in another part of the building in entertaining to luncheon the Prince of Wales and the Elder Brethren of Trinity House. It would probably be strange to many, he said, to learn that an orthodox Red Cross Society had not existed in England until the present movement was inaugurated in July last. The Association, which called itself the British National Society for Aid to the Sick and Wounded in War, owed its inception to the great and gallant soldier, Lord Wantage, who had served in the Crimea, and was witness to the sufferings of our soldiers and sailors in that war caused by the want of foresight of what modern war was and would be. After the Crimean war numerous campaigns took place—the American Civil war, the Franco-Italian campaign, the war in Denmark, and the great struggle between Prussia and Austria. After those campaigns the Geneva Society was founded, and rules were laid down protecting those who gave medical assistance from the results of warfare. Shortly afterwards that great struggle took place between France and Germany. It was on that occasion that Lord Wantage started the National Aid Society, and the large sums which a generous public contributed enabled the Society to alleviate the sufferings of the combatants in that campaign, which lasted for about a year. After that the National Society rendered assistance to both combatants in many campaigns, but it could not be said that that assistance was greatly welcomed. There was a feeling that the presence of amateurs in the field was undesirable; they were looked upon as newspaper spies and critics anxious to detect defects in the armour. Just before the South African war Lord Lansdowne, as Secretary for War, originated the Red Cross Council, over which Lord Wantage presided, and various societies were affiliated. When the war broke out the assumption was that hospitals and comforts would only be necessary for 60,000 men. Before long, however, we had a quarter of a million men in the field, and at one time the troops numbered 300,000. He mentioned that fact because of the criticisms which had been levelled against the medical arrangements in the field, and the extra medical comforts and arrangements which charitable organisations tried to provide. Eventually the difficulties encountered were overcome, and he would remind them that at the relief of Ladysmith, of Kimberley, and of Mafeking they entered almost simultaneously with the troops, and that there never was a case of a sick or wounded soldier but the relations were able to communicate with him through the National Aid Society. One lesson that war had taught the nation, that, however desirous they might be of settling differences by diplomacy, it was necessary to mature preparations beforehand. There was a great difference between the British Red Cross Society and Red Cross Societies abroad. The British Society, as at present constituted, was only intended to act in time of war. When a disaster or an epidemic occurred in England the local authorities were amply able to act, and did not require assistance. Another difference was that here the Navy and the Army were composed of volunteers, and their wants were, or ought to be, amply provided for by the First Lord of the Admiralty and the Secretary of State for War. The preparations which the British Red Cross Society would have to make were only necessary in case of war, and would require large sums of money and involve a great deal of organisation of the details of which he would leave Lord Tweedmouth, Lord Methuen, and Sir Frederick Treves to speak.

Lord Tweedmouth (First Lord of the Admiralty) moved that this meeting, assembled under the presidency of Lord Rothschild, heartily approves of the objects of the British Red Cross Society, and wishes it every success under its new organisation. He said he thought there was no idea and no object that impressed itself more on the patriotic and benevolent men and women of every country in the civilised world than that it was the duty of the general public to do all that could be done to mitigate the horrors of war and to relieve the sufferings which the soldiers and sailors were subject to in time of war. He thought such a feeling had prevailed as long as civilised nations had existed, but it was stronger now than ever since the destructiveness of war had been so greatly intensified by modern appliances. The catastrophes of modern warfare were terrible to think of. They had had in the recent Russo-Japanese war experiences of the frightful amount of injury inflicted on those engaged. We too had had experience during the South African war. It might be said, Why should one of those who represented the defensive services of the country ask aid from the public in work which should fall on the Government Departments which had the care of the defensive services? He would say on behalf of both services that they did take care, and had constantly in mind the consideration of the most terrible problem of dealing with the sick and wounded in time of war. If they went to Haslar, to Devonport, to Chatham, and to Queenstown they would see how modern appliances had been adopted. If they looked at H.M.'s ships they would see what preparations were made for the comfort and safety of the men who served in them. At Devonport and at Portsmouth they would see vast accumulations of medical stores. Everything that mortal man could devise was and would be done. But it was impossible to provide for all the contingencies of war; and what, as representing the Admiralty, he pressed on that Society was not that they should be relieved of that duty which they accepted and acknowledged, but that their work should be supplemented by their efforts and monetary help so that they might meet the necessities and exigencies which must arise in modern warfare. It was impossible to keep up sufficient personnel in time of peace for all the requirements in time of war. They must look to such a society to supplement their preparations and their provisions. At the time of the South African war it was not owing to any want of will that they were perhaps deficient in medical service. The public came nobly to their aid, and the story of the hospitals put into the field by private effort would ever be memorable in their history. But there was a certain amount of overlapping and doubling of effort, and it was felt that there should be some central organisation to organise and supervise the work; which should in time of peace collect the names of those willing to serve; should collect dressings and ambulances, and should take the lead in putting the hospital organisation into the field when necessity arose, with tact and energy. The other societies, the St. John's Ambulance and the St. Andrew's, were willing to render every assistance, but it was absolutely necessary that the public at large should support the movement by giving all the moral and financial aid necessary to bring it to a satisfactory conclusion. He would remind them that the objects of the Red Cross Society were very dear to the heart of our gracious Queen, and that from the beginning she had taken the greatest interest in its operations. There was no more worthy object for the help of the country at large. He put forward its claims, not on the ground of charity, but as a national duty. They who represented the Navy and the Army were only too glad to have help; they recognised they were bound to give all the aid they could, but that they could not do all that was necessary, and they appealed to the larger public to supplement their work and make it perfect. (Applause.)

Lord Methuen seconded the resolution. He urged that that Association should be extended to all countries and to the large towns and cities in the Empire. It was a great thing to have a trained personnel to rely on, and there was no difficulty in men having an opportunity of learning the work of the hospitals. Another matter in which the Society could help was in the study of sanitation, in which we were much behind the Japanese. Speaking as Chairman of King's College Hospital, he felt that there was nothing so useful to soldiers as to get in touch with the medical profession, and so have their minds opened.

The resolution was agreed to.

Sir Frederick Treves rose to propose a vote of thanks to the Lord Mayor for the use of the Mansion House, and said that before speaking to the resolution he was commissioned by the Prince of Wales to convey to the meeting the following message: "I wish success to the British Red Cross Society, and am impressed with the necessity of making preparations for war in time of peace. The Society is one whose welfare Her Majesty the Queen has much at heart, and as its object is to convey practical sympathy to our soldiers and sailors, when engaged in war, it should meet with the support of all classes of the community. Much has been done in the past: much generous aid has been forthcoming from the colonies, and I hope that in time a branch of the British Red Cross Society will be formed in every part of the Empire." (Applause.) The conspicuous note of that message, said Sir Frederick, was the necessity for preparedness for war in time of peace. The heart of the country was kindly, and when need arose offers of help and money poured in, but there was then much overlapping and waste of energy. He could not help recalling the last terrible sentence of the great history of the Peninsular war: "And thus ended the great war; and the veterans were soon forgotten." The cynicism of that phrase was perfect: "soon forgotten." It was greatly to be hoped that the lessons as to the dangers of unpreparedness we had learned so hardly in the late war would not be "soon forgotten." Then the message alluded to the interest taken in the Society by the Queen. He need not remind them that the reconstituted Society was inaugurated by Her Majesty; but it was not generally known that she saw the minutes of every meeting, and commented and advised on them, and that no departure was taken in connection with their work without consulting the Queen. It was indeed essentially a woman's movement, and if only one woman in a hundred would take as much interest in it as the Queen devoted to it, its success would be beyond any kind of question. (Applause.) The Red Cross Society meant the holding out the hand of sympathy to the men at the seat of war, and bringing to them the touch of home. His mind went to Scutari with its memories of Florence Nightingale—"the lady with the lamp." The hospital still stood as it did 50 years ago—a miserable squalid place—and let them think what it meant to our soldiers and sailors in those wards to realise that "the lady with the lamp" had been sent to their succour by the people of this country. Outside, too, was the burial-ground—a piece of rural England, planted with English trees, by the very edge of the sea. That was a picture of what was meant by the Red Cross Society; and there lay the chief matron, the nurses, and the doctors, for there they all died. That Society, he could not help feeling, was fitly represented by the patient figure of "the lady with the lamp." (Applause.) Concluding, he said they would, he knew, give their hearty thanks to the Lord Mayor (who at that moment entered the room), and he need not remind them that a Society which started under the ægis of the Mansion House started well. (Applause.)

The vote was carried by acclamation, as was also one to Lord Rothschild for his conduct in the chair.

